# National epidemiology of initial and recurrent *Clostridium difficile* infection in the Veterans Health Administration from 2003 to 2014

**DOI:** 10.1371/journal.pone.0189227

**Published:** 2017-12-07

**Authors:** Kelly R. Reveles, Kenneth A. Lawson, Eric M. Mortensen, Mary Jo V. Pugh, Jim M. Koeller, Jacqueline R. Argamany, Christopher R. Frei

**Affiliations:** 1 College of Pharmacy, The University of Texas at Austin, Austin, Texas, United States of America; 2 Pharmacotherapy Education and Research Center, UT Health San Antonio, San Antonio, Texas, United States of America; 3 South Texas Veterans Health Care System, San Antonio, Texas, United States of America; 4 Department of Internal Medicine and Clinical Sciences, University of Texas Southwestern Medical Center, Dallas, Texas, United States of America; 5 VA North Texas Health Care System, Dallas, Texas, United States of America; 6 Department of Epidemiology and Biostatistics, UT Health San Antonio, San Antonio, Texas, United States of America; Cleveland Clinic, UNITED STATES

## Abstract

**Introduction:**

Prior studies demonstrated marked increases in *Clostridium difficile* infection (CDI) in the United States (U.S.) in recent years. The objective of this study was to describe the epidemiology of initial and recurrent CDI in a national Veterans Health Administration (VHA) cohort over a 12-year period.

**Methods:**

This was a retrospective cohort study of all adult VHA beneficiaries with CDI (ICD-9-CM code 008.45) plus a positive CDI stool test between October 1, 2002 and September 30, 2014. Data were obtained from the VA Informatics and Computing Infrastructure. Recurrence was defined as a second ICD-9-CM code plus a new course of CDI therapy following a minimum three-day gap after the initial therapy was completed. CDI incidence and outcomes were presented descriptively and longitudinally.

**Results:**

Overall, 30,326 patients met study inclusion criteria. CDI incidence increased from FY 2003 (1.6 per 10,000) to FY 2013 (5.1 per 10,000). Thereafter, CDI incidence decreased through FY 2014 (4.6 per 10,000). A total of 5,011 patients (17%) experienced a first recurrence and, of those, 1,713 (34%) experienced a second recurrence. Recurrence incidence increased 10-fold over the study period, from (0.1 per 10,000) in FY 2003, to (1.0 per 10,000) in FY 2014. Overall, 30-day mortality and median hospital length of stay (LOS) decreased among initial episodes over the study period. Mortality was higher for initial episodes (21%) compared to first recurrences (11%) and second recurrences (7%). Median hospital LOS was longer for first episodes (13 days) compared to first (9 days) and second recurrences (8 days).

**Conclusions:**

Initial and recurrent CDI episodes increased among veterans over a 12-year period. Outcomes, such as mortality and hospital LOS improved in recent years; both of these outcomes are worse for initial CDI episodes than recurrent episodes.

## Introduction

*Clostridium difficile* is the most prevalent pathogen among all healthcare-associated infections [1]. This Gram-positive, anaerobic bacterium can colonize the human gut, typically following health care contact and exposure to agents that disrupt the normal gut microbiota, like antibiotics. Patients may then develop a toxin-mediated intestinal disease, *Clostridium difficile* infection (CDI). Disease recurrence is a common and costly outcome of CDI. Approximately 14% to 26% of individuals experience CDI recurrence despite successful treatment of the initial episode [2–5]. In those patients who have already experienced one recurrence, the risk of additional recurrences may be as high as 65% [6].

National epidemiological investigations have demonstrated marked increases in CDI in the United States (U.S.) in recent years. Two recent studies demonstrated that CDI incidence nearly doubled in U.S. community hospitals in the first decade of the twenty-first century [7, 8]. Until recently, these nationally-representative CDI studies excluded federal facilities, like the Veterans Health Administration (VHA). Evans et al. [9, 10] described the burden of CDI among veterans and found a decline in the CDI incidence in VHA acute care facilities nationally from 2010 to 2015. Our study aims to supplement these findings by describing longitudinal trends in CDI incidence and health outcomes in outpatient and inpatient settings for first episodes, as well as first and second recurrences, over a 12-year period.

## Methods

### Study design

This was a national, retrospective cohort study of all CDI patients receiving care at any of the approximately 150 VHA hospitals and 820 VHA clinics in the U.S. Data for this study were obtained from the VA Informatics and Computing Infrastructure (VINCI), which includes administrative, clinical, laboratory, and pharmacy data repositories which are linked using unique patient identifiers. All data collection and analyses were performed at the South Texas Veterans Health Care System, Audie L. Murphy Veterans Affairs (VA) Hospital, San Antonio, TX. The Institutional Review Boards at UT Health San Antonio and the South Texas Veterans Health Care System Research and Development Committee approved this study and waived the need for informed consent.

### Study population

The initial cohort was created by identifying all adult patients (age 18 to 89 years) who had any inpatient or outpatient *International Classification of Diseases*, *9th Revision*, *Clinical Modification* (ICD-9-CM) code for CDI (008.45) plus any positive stool test (e.g., glutamate dehydrogenase, enzyme immunoassay, polymerase chain reaction) for CDI during the visit or within 7 days of the visit from October 1, 2002 through September 30, 2014. We limited our cohort to first-episode CDI patients only. This was accomplished by excluding those patients with an ICD-9-CM code for CDI (008.45) in the year prior to study inclusion.

### Study definitions

A first episode was defined as described above. A first recurrence was defined as a second outpatient or inpatient visit during which a patient received an ICD-9-CM code for CDI, plus a minimum 3-day gap between the visit and the end of active CDI therapy (e.g., metronidazole, oral vancomycin, fidaxomicin, rifaximin, nitazoxanide, probiotics) for the initial episode. For those in whom CDI therapy was not listed in the medical record, the gap was defined from the day of the outpatient encounter or hospital discharge to a second outpatient or inpatient visit. A second recurrence was defined in the same manner as the first, but using the third CDI diagnosis over the cohort period.

Patient demographics included age during the initial CDI episode, sex, race, and ethnicity. Sex, race, and Hispanic ethnicity were defined as the most frequent reporting of each characteristic over the study period. Principal CDI was defined as ICD-9-CM code 008.45 in the first position. This often indicates that CDI was the primary contributor to hospitalization. Secondary CDI was defined as ICD-9-CM code 008.45 in any position except first. CDI was also characterized by type. Community-onset CDI (CO-CDI) was defined based upon the presence of CDI therapy initiated in the outpatient setting or on days 1 or 2 of hospitalization. Community-onset, healthcare facility-associated CDI (CO-HCFA-CDI) was defined the same way, with the addition of a hospitalization in the prior 90 days. Lastly, healthcare facility-onset CDI was defined as CDI therapy beginning on day 3 or later of hospitalization.

We collected Charlson comorbidities and other relevant diagnoses, as defined by ICD-9-CM codes, in the year prior to the first CDI episode (S1 Table). We also calculated the Charlson comorbidity score as modified by Deyo et al. [11]. In addition, we collected other infections (as defined by ICD-9-CM code, S1 Table) that occurred during a CDI episode (between CDI episode start date and end of CDI therapy), including: bacteremia, pneumonia, skin infection, intra-abdominal infection, urinary tract infection, device-related infection, endocarditis, and acute respiratory infection. Other markers of CDI severity that occurred during a CDI encounter were also captured, including ICU admission, sepsis/septicemia, shock, acute renal failure, megacolon, prolonged ileus, perforated intestine, colectomy, white blood cell count (WBC), C-reactive protein (CRP), serum creatinine (SCr), and albumin.

Prior and concomitant non-CDI antibiotics (excludes oral vancomycin, metronidazole, fidaxomicin, rifaximin, and nitazoxanide), non-CDI high-risk antibiotics (third and fourth generation cephalosporins, fluoroquinolones, and clindamycin), gastric acid-suppressing (GAS) drugs (antacids, H2 blockers, proton pump inhibitors), anti-diarrhea medications, narcotics, and bowel prep medications were also collected. Prior use was defined as any use in the 90 days prior to a CDI encounter. Concomitant use was defined as any use during or within 60 days following a CDI episode.

CDI incidence was calculated as CDI episodes per 10,000 VHA enrollees. All-cause mortality was defined as death within the 30, 60, or 90 days following CDI treatment discontinuation. For patients who were hospitalized with CDI, hospital LOS was defined as date of discharge minus date of admission plus one day.

### Data and statistical analyses

Data extraction and variable creation were conducted using SAS Version 9.2^®^ (SAS Corp., Cary, NC, USA). All other data and statistical analyses were conducted using JMP 13.0^®^ (SAS Corp., Cary, NC, USA).

All independent and dependent variables were first presented descriptively. For baseline characteristics (e.g., sex, race, ethnicity), we included a missing category. Other variables that were absent from the medical chart (e.g., comorbidities) were assumed to have not occurred [12, 13].

We described the epidemiology of CDI first episodes from fiscal year (FY) 2003 to FY 2014. Fiscal year served as the independent variable. The dependent variables included CDI incidence, first and second recurrence, 30-, 60-, and 90-day patient mortality, and hospital LOS. Categorical variables were presented as the proportion of patients experiencing each outcome. Hospital LOS was presented as the median (interquartile range). Mortality was compared between episode types using the chi-square test and hospital LOS was compared using the Wilcoxon Signed rank test. We also compared 30-day mortality between first episodes and first recurrences using a multivariable model that included all baseline characteristics listed in Table 1, plus receipt of metronidazole or vancomycin CDI therapy, as characterized during each episode.

**Table 1:** Baseline characteristics.

Characteristic	n = 30,326
Age (years), median (IQR)	67 (60–78)
Male sex, %	95.9
Race & ethnicity, %	
Non-Hispanic White	66.2
Non-Hispanic Black	21.1
Hispanic	5.4
Other	4.4
Missing	2.9
Principal CDI diagnosis, %	28.1
CDI type, %	
CA-CDI	19.2
CO-HCFA-CDI	20.6
HCFO-CDI	60.2
Comorbidities, %	
Hypertension	77.6
Dyslipidemia	54.6
Obesity	16.5
Myocardial infarction	11.3
Congestive heart failure	26.5
Peripheral vascular disease	19.4
Cerebrovascular disease	19.7
Dementia	3.7
COPD	37.9
Rheumatologic disease	2.8
Peptic ulcer disease	4.8
Liver disease	7.2
Diabetes	41.1
Hemiplegia or paraplegia	4.2
Renal disease	28.3
Cancer	28.9
HIV/AIDS	1.9
GERD	27.0
Transplant	2.0
Inflammatory bowel disease	2.4
Irritable bowel syndrome	1.1
Charlson score, median (IQR)	3 (2–6)
Concomitant infections, %	
Bacteremia	7.0
Pneumonia	23.0
Skin infection	10.9
Intra-abdominal infection	6.0
Device-related infection	3.3
Acute respiratory infection	3.4
Endocarditis	1.0
Urinary tract infection	1.8
CDI severity indicators, %	
ICU admission	1.7
Sepsis/septicemia	17.7
Shock	5.1
Acute renal failure	30.8
Megacolon	0.3
Prolonged ileus	4.1
Perforated intestine	0.5
WBC ≥15,000 cells/μL	39.2
CRP ≥160 mg/L	1.7
Albumin <2.5 g/dL	32.8
SCr >1.5 mg/dL	24.3
Colectomy	0.1
Medications, %	
Prior antibiotics	56.7
Prior high-risk antibiotics	38.4
Prior GAS drugs	56.9
Prior narcotics	38.8
Prior anti-diarrheals	7.6
Prior bowel prep	15.7
Concomitant antibiotics	75.2
Concomitant high-risk antibiotics	52.4
Concomitant GAS drugs	79.3
Concomitant narcotics	51.1
Concomitant anti-diarrheals	11.3
Concomitant bowel prep	19.5

AIDS = acquired immune deficiency syndrome; CDI = *Clostridium difficile* infection; CA-CDI-community-associated CDI; CO-HCFA-CDI = community-onset, healthcare facility-onset CDI; COPD = chronic obstructive pulmonary disease; CRP = C-reactive protein; GAS = gastric acid-suppressing; GERD = gastroesophageal reflux disease; HCFO-CDI = healthcare facility-onset CDI; HIV = human immunodeficiency syndrome; ICU = intensive care unit; IQR = interquartile range; SCr = serum creatinine; WBC = white blood cells.

## Results

### Baseline characteristics

Overall, 30,326 unique patients with a first CDI episode met study inclusion criteria. Table 1 describes the patients’ baseline characteristics. Patients were predominately elderly (median age 67 years), male (96%), and White (67%). CDI was listed as the secondary diagnosis for the majority of patients (72%). The median (IQR) Charlson comorbidity score was 3 (2–6). The most common comorbidities included: hypertension (78%), dyslipidemia (55%), diabetes (41%), COPD (38%), and cancer (29%). Patients also commonly presented with concomitant infectious diagnoses, including pneumonia (23%) and skin infections (11%), concomitant antibiotics (75%), and GAS drugs (79%).

### CDI initial episode and recurrence incidence

**Fig 1:**
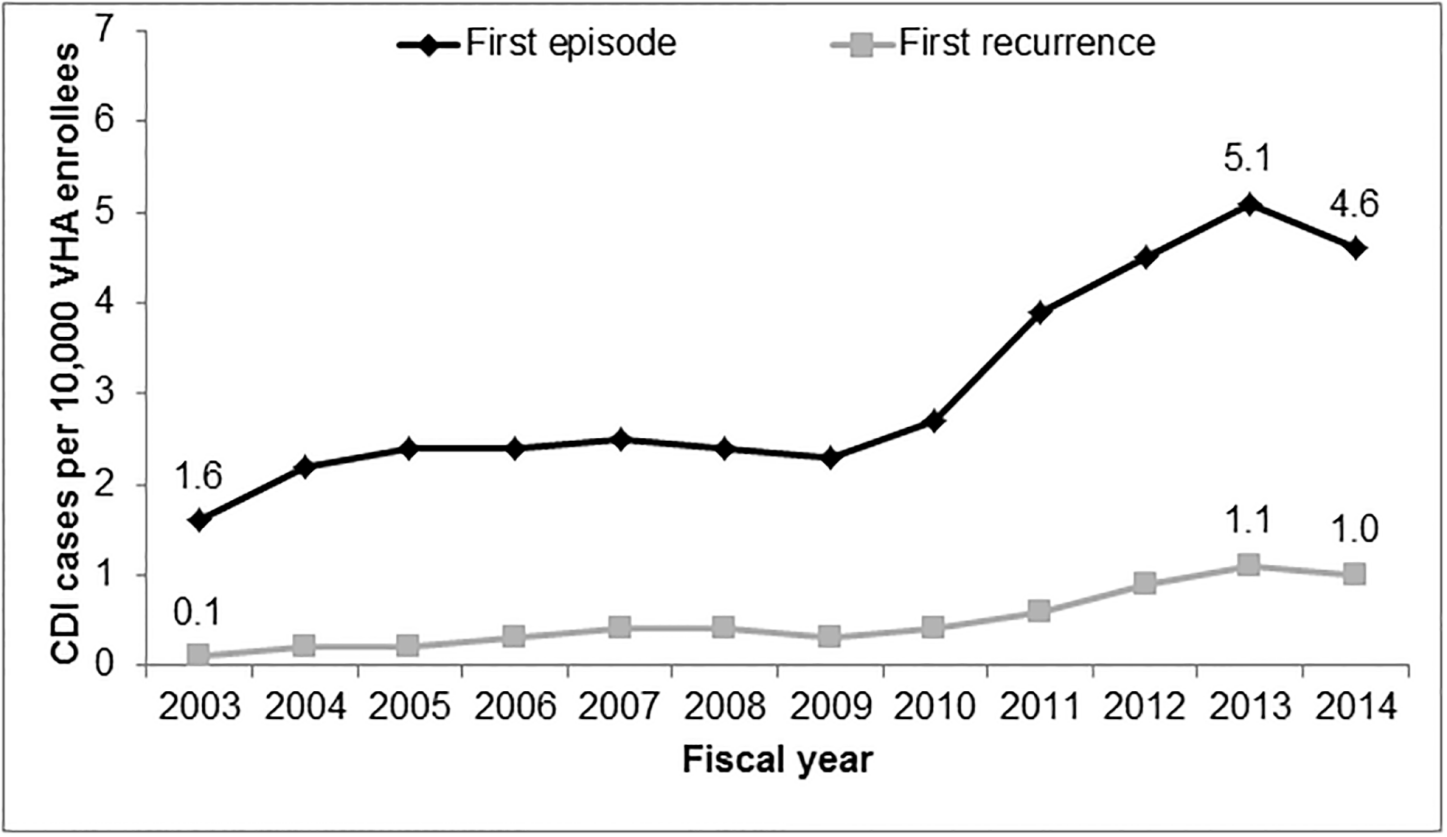
CDI incidence from FY 2003 to FY 2014, n = 30,326.

The overall incidence of CDI over the study period was 3.1 per 10,000 VHA enrollees. CDI incidence increased from FY 2003 (1.6 per 10,000) to FY 2013 (5.1 per 10,000) (Fig 1). Thereafter, CDI incidence decreased through FY 2014 (4.6 per 10,000).

Overall, 5,011 patients experienced a CDI first recurrence over the study period. This represents 17% of the total CDI cohort. Of these patients, 1,713 experienced a second recurrence, representing 34% of patients who had a first recurrence. The proportion of patients with a secondary CDI diagnosis was 97.3% for first recurrences and 99.2% for second recurrences. The overall incidence of CDI first recurrences over the study period was 0.5 per 10,000 VHA enrollees. The incidence increased 10-fold from FY 2003 (0.1 per 10,000) to FY 2014 (1.0 per 10,000).

### Mortality

**Fig 2:**
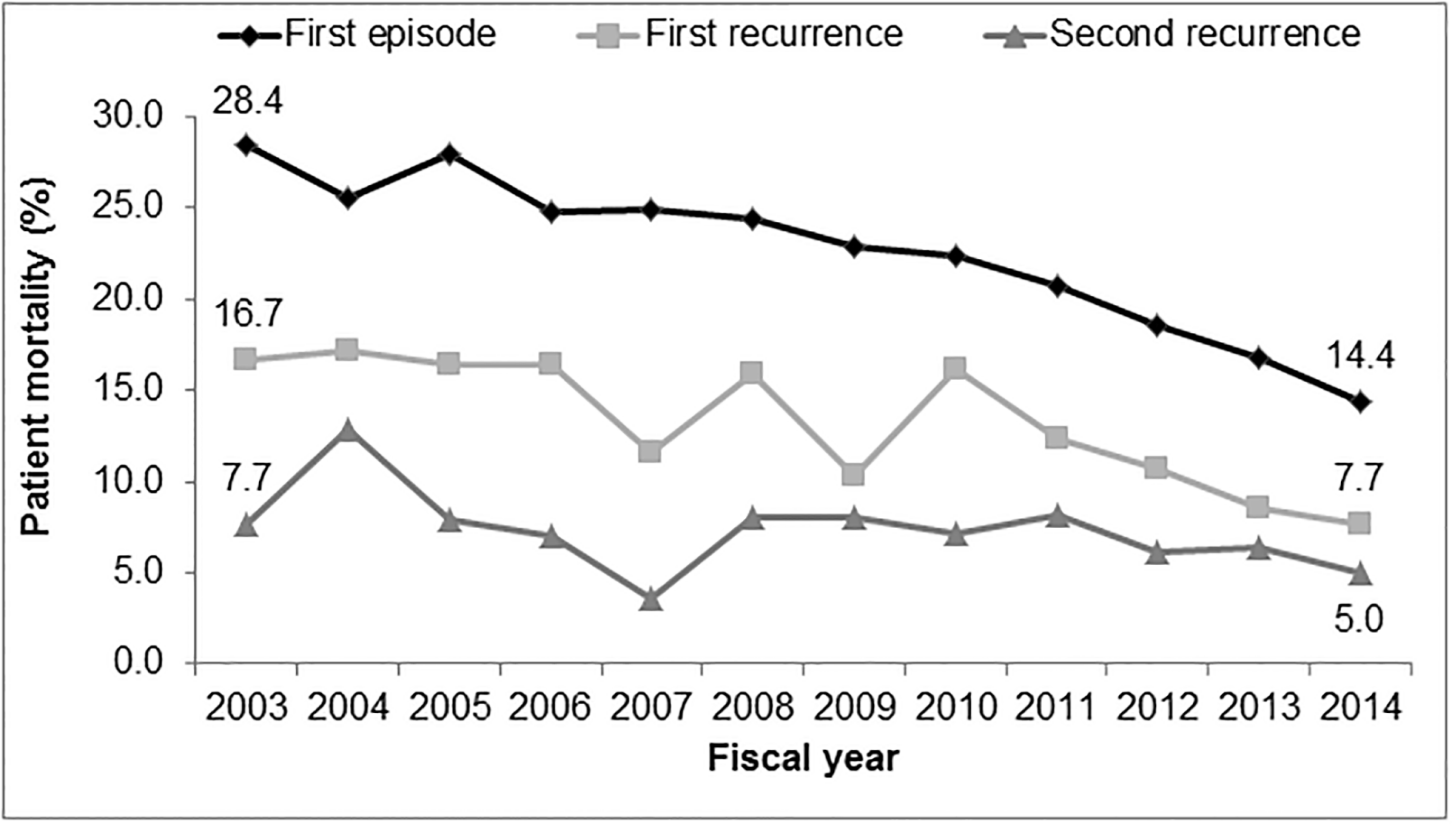
30-day mortality among patients with CDI from FY 2003 to FY 2014, n = 30,326.

Overall, 30, 60, and 90-day mortalities among first episode CDI patients were 21%, 25%, and 28%, respectively. For first recurrences, 30, 60, and 90-day mortalities were 11%, 15%, and 17%, respectively. For second recurrences, 30, 60, and 90-day mortalities were 7%, 9%, and 11%, respectively. Thirty-, 60-, and 90-day mortalities among CDI first recurrence patients were significantly lower than for first episode patients (p<0.0001 for all). First episodes were independently associated with 30-day mortality compared to first recurrences (odds ratio 3.59, 95% CI 2.43–5.28). Thirty, 60, and 90-day mortality among patients with a second CDI recurrence were significantly lower compared to patients with a first episode (p<0.0001) or first recurrence (p<0.0001 for all). Mortality decreased over the study period for initial episodes, first recurrences, and second recurrences (Fig 2).

### Hospital LOS

**Fig 3:**
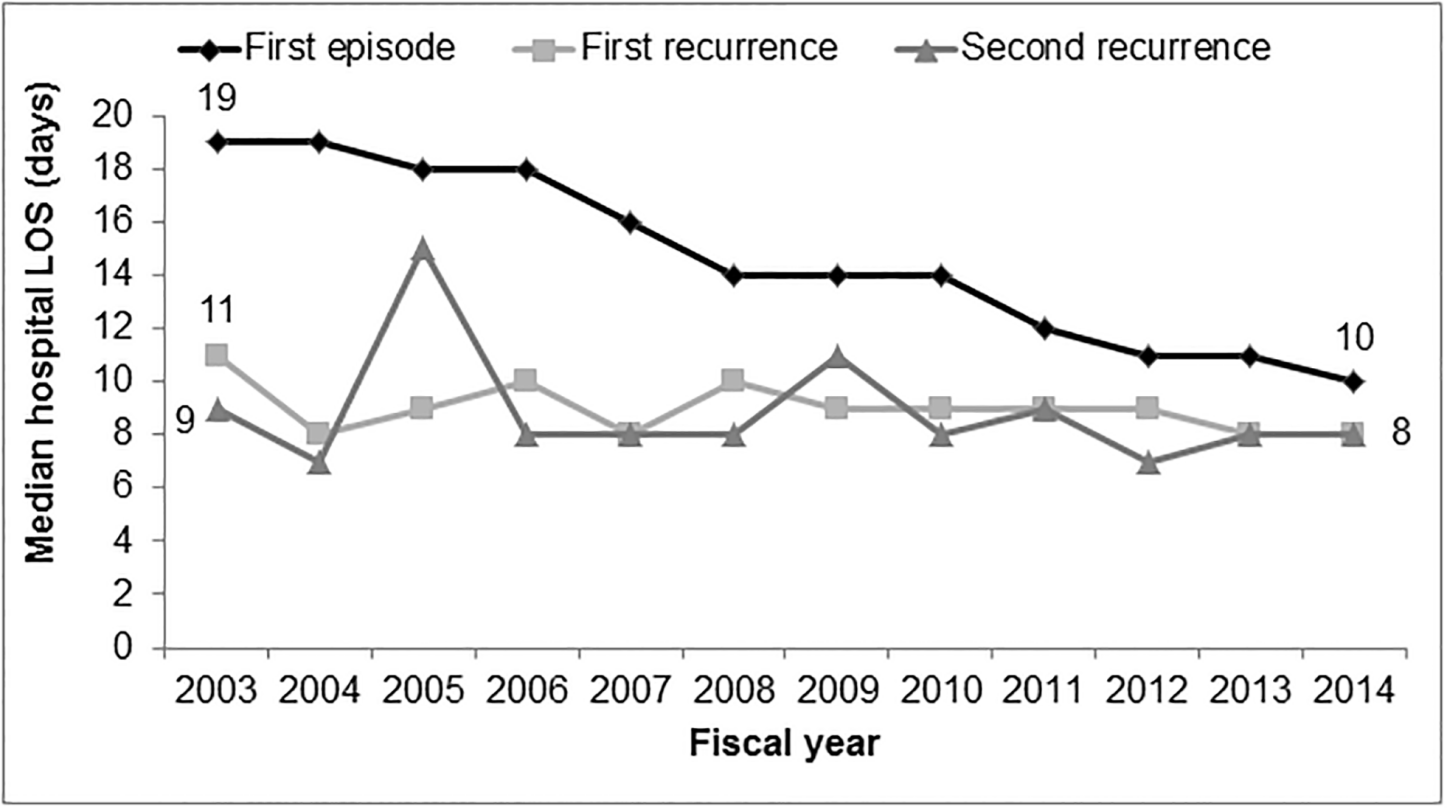
Median hospital LOS among patients with CDI from FY 2003 to FY 2014, n = 30,326.

Approximately 93% of CDI patients in our cohort were hospitalized, whereas 7% of patients received care in the outpatient setting only. The median (IQR) hospital LOS for first episodes was 13 (7–30) days. The median (IQR) hospital LOS among patients with a first recurrence was 9 (5–19) days. This represents a reduction in median hospital LOS of 4 days compared to the first CDI episode (p<0.0001). The median (IQR) hospital LOS among patients with a second CDI recurrence was 8 (5–16) days, which was also significantly shorter than initial episodes (p = 0.0005), but not first recurrences (0.1220). Median hospital LOS decreased for first episodes, but remained relatively stable over the study period for first and second recurrences (Fig 3).

## Discussion

This study documents the burden of CDI among adult veterans in the U.S. in recent years. Despite increases in CDI initial and recurrent episodes, we found marked improvements in relevant health outcomes, including mortality and hospital LOS, throughout the study period. This is one of the first studies to demonstrate improved outcomes in a national sample of CDI patients in the U.S. Our study is strengthened by the national scope, comprehensive computerized data available through the VHA, the ability to study inpatient and outpatient occurrences of CDI, and the separation of initial and recurrent episodes.

Prior national studies utilizing the U.S. Nationwide Inpatient Sample and National Hospital Discharge Surveys found dramatic increases in CDI in community hospitals in recent years. Specifically, these studies demonstrated a near doubling of CDI incidence between approximately 2001 and 2010 [7, 8]. CDI incidence seems to have also increased dramatically among veterans compared to the general population. A prior study by Young-Xu et al. [14] reported an increase of 19% in CDI episodes from 2009 to 2013 with a stabilization of rates from 2011 to 2013 in the VHA population. Our results are in-line with this prior study; however, differences in incidence rates can be attributed to the longitudinal nature of our study, in which we were able to separate initial episodes from recurrences. Other studies by Evans et al. [9, 10] noted a significant decline in CDI in VHA acute care facilities from 2010 to 2015. These robust analyses utilized clinically confirmed CDI, but limited only to hospital inpatients, which helps explain differences in trends in recent years.

Changing diagnostics could help explain CDI incidence trends. Nucleic acid amplification tests (NAAT), which are more sensitive than traditional *C*. *difficile* stool tests (e.g., toxin A/B enzyme immunoassay) [15], have been used more frequently in clinical practice in recent years. Evans et al. reported that NAAT use increased in VHA acute care facilities from 32.5% in 2010 to 81.1% in 2015 [10]. Increased use of NAAT has been previously associated with increased CDI incidence [16].

Despite improvements in recent years, mortality remains a great concern for patients with CDI. Our study demonstrated that nearly one-fifth of all veterans with CDI die within 90 days of diagnosis. Prior studies have found that all-cause mortality among hospitalized CDI patients ranges from 14% to 25% at 30 days, 17% to 22% at 60 days, and 23% to 29% at 90 days following CDI diagnosis [17–24]. Evans et al. [10] recently reported a 30-day all-cause mortality rate of 10.6% for VHA patients with clinically confirmed hospital-onset CDI between 2012 and 2015. Among critically-ill patients, 30-day mortality is substantially higher (37%) [24, 25], while mortality among patients with community-onset CDI is much lower (3% to 17%) [23]. Importantly, our study found that mortality among veterans with CDI decreased in recent years. This is in contrast to other studies that demonstrated dramatic increases in CDI mortality in the U.S. and Canada from the late 1990s to the early 2000s [19, 26–28]. More recent studies have demonstrated lower mortality among hospitalized patients with CDI. A national study in England demonstrated that since its peak in 2007, mortality from CDI fell over 80% [29]. Smaller studies in the U.S. have noted similar reductions. Mermel et al. [30] reported a nearly 50% reduction in mortality among CDI patients from 2010 to 2011 in a large hospital in Rhode Island, following implementation of a multidisciplinary CDI intervention program.

Our study found a substantially lower mortality rate and shorter hospital LOS among patients with recurrent CDI compared to first episodes. First, survival bias could influence mortality rates in recurrences, as those patients who survived the initial episode are more likely to survive subsequent episodes. We also suspect that recurrence is associated with earlier disease detection, leading to earlier therapeutic interventions. Additionally, host adaptive immune response stimulated by the initial episode may limit severity in recurrences, [31] thus resulting in lower mortality rates and shorter hospital stays. The shorter LOS in recurrences might also indicate a greater propensity for treating recurrences in the outpatient setting or earlier transition of care. In a study following 372 primary cases of CDI in a large hospital, Aitken et al. [32] found that 45% of patients with a CDI recurrence were treated in the outpatient setting without subsequent re-hospitalization. Additionally, patients with multiple recurrences were more frequently treated in the outpatient setting.

In addition to poor health outcomes, CDI contributes to substantial health care resource utilization. In a review of four large studies of CDI in hospitalized patients, Gabriel et al. found the mean attributable costs ranged from $8,426 to $11,228 and the mean LOS increased by 5–16 days [33]. Other studies have estimated the median LOS for hospitalized patients with CDI to be 8 to 13 days [4, 7, 8, 34–36]. In critically-ill patients, median LOS is substantially longer (28 days) [24]. CDI results in considerably longer hospital LOS compared to non-CDI patients. Lucado et al. [8] demonstrated that mean hospital LOS among patients with CDI was 13 days, compared to 5 days for hospitalized patients without CDI. This is likely due to the need for antibiotic therapy and close monitoring of patients with CDI. Furthermore, CDI can complicate comorbid conditions, and result in the need for additional hospital resources. The decline in hospital LOS over time in our study may reflect a greater push for outpatient therapy in more recent years, as this can greatly reduce costs associated with treating CDI. CDI therapy is predominately oral; therefore, the transition from inpatient to outpatient therapy can typically be achieved quickly, especially among patients with mild disease.

Consistent with prior studies in community hospitals, CDI recurrence is a major public health problem in the VHA. Approximately 17% of veterans with CDI experienced disease recurrence and the incidence increased markedly over the study period. Prior studies have estimated that CDI recurrence occurs at a rate of 7% to 26% at 30 days, 10% to 26% at 60 days, and 16% to 28% at 90 days [4, 17, 21, 35–44]. Few studies have evaluated CDI recurrences over time. In 2005, Pepin et al. published the first study describing CDI recurrences longitudinally in a cohort of CDI patients in Quebec, Canada.[45] This study demonstrated that CDI recurrence more than doubled, from 20.8% in 1991–2002 to 47.2% in 2003–2004 [45].

CDI continues to be a major public health problem in the U.S., and specifically in the veteran population. Aggressive clinical and public health initiatives should be implemented to reduce the incidence of these infections in hospitals and the community. Antibiotic stewardship programs (ASPs) play a critical role in improving prescribing practices and promoting more judicious antimicrobial utilization. Numerous reports have shown a reduction in CDI incidence and decreased hospital LOS following implementation of ASPs or specific antimicrobial restriction policies [46–53]. ASPs have been a priority among national health care systems, including the VHA. As of 2012, 64% of VHA facilities had an antimicrobial stewardship policy in existence or in development [54]. Since then, a national VHA antimicrobial stewardship policy, VHA Directive 1031, was developed to establish stewardship programs in all VHA facilities by July 2014 [55].

This study has potential limitations. First, we utilized a retrospective cohort study design that includes data collection from electronic medical records. The use of ICD-9-CM codes to identify CDI cannot be considered equivalent to medical chart review or prospective data collection; however, a prior study demonstrated relatively high sensitivity (78%) and specificity (99.7%) of the CDI code compared to microbiological data [56]. We aimed to improve our case definition to limit misclassification bias by only including those patients who had a positive CDI stool test listed in the medical chart. Furthermore, ICD-9-CM codes were used to define other comorbidities, including concomitant infections. These codes have variable accuracy for identifying infections and could result in misclassification [57, 58]. Next, the use of an ICD-9-CM code in the first position is not consistently used across all VHA facilities to indicate a primary diagnosis; therefore, our classifications of principal and secondary CDI might be imperfect. Similarly, the large sample size precluded our ability to confirm CDI recurrence based on clinical symptoms following cure of the initial episode, as is the most common recurrence definition [59, 60]. Therefore, we relied on a receipt of a second course of CDI therapy to indicate recurrence. We chose a three-day gap between regimens to limit misclassification of recurrences as treatment failures, although this definition has not been validated. Not all patients had CDI therapy listed in the medical record. It is not known if these patients were untreated or simply filled their prescription outside of the VHA system. Other factors could have influenced CDI incidence rates in this study. The increased use of NAATs and use of motility agents (e.g., laxatives) could generate more inappropriate diagnoses of patients who are simply colonized with *C*. *difficile*. Next, prior studies have used different time frames (60 days to 10 years) to limit their cohort to first episode patients only [4, 37]. Most CDI recurrences occur within 60 to 90 days of treatment discontinuation for the initial episode [2]; few recurrences will occur after this time frame. We felt that one year was sufficient to limit our cohort to first episode CDI patients only; however, this definition has not been validated. Some CDI episodes could have been missed, as veterans could have been treated at non-VHA facilities, especially those with severe CDI who might have needed a higher level of care. The use of VHA enrollees was selected as the incidence denominator for consistency between inpatients and outpatients; however, other measures of burden, such as hospital bed days, may more accurately describe hospital-onset CDI incidence. Finally, our predominately elderly, male, veteran CDI population might not be representative of all CDI populations, thus, potentially limiting the generalizability of our epidemiological findings to other settings.

## Conclusions

Initial and recurrent CDI episodes increased among veterans over a 12-year period; the most recent year suggests these infections may finally be on the decline. Outcomes such as mortality and hospital LOS have improved in more recent years; both of these outcomes are worse for initial CDI episodes than recurrent episodes. CDI continues to be an important public health problem in the VHA, and further efforts are needed to prevent and treat initial and recurrent infections.

## Supporting information

S1 Table Study comorbidity definitions. (DOCX)

S1 File Limited dataset. (CSV)
