# Supplementary material for: National epidemiology of initial and recurrent Clostridium difficile infection in the Veterans Health Administration from 2003 to 2014
Source: PLoS One. 2017 Dec 7;12(12):e0189227. doi: 10.1371/journal.pone.0189227 (PMC5720754; doi:10.1371/journal.pone.0189227)
Supplement: S1 Table — (DOCX) [file pone.0189227.s001.docx]

| **Comorbidity** | **ICD-9-CM code(s)** |
| --- | --- |
| **Comorbidities in year prior to CDI episode** | |
| Hypertension | 401-405 |
| Dyslipidemia | 272 |
| Obesity | 278 |
| Myocardial infarction | 410, 412 |
| Congestive heart failure | 428 |
| Peripheral vascular disease | 441, 443.9, 785.4, V43.4 |
| Cerebrovascular disease | 430-438 |
| Dementia | 290 |
| COPD | 490-496, 500-505, 506.4 |
| Rheumatologic disease | 710.0-710.1, 710.4, 714.0-714.2, 714.81, 725 |
| Peptic ulcer disease | 531.0-531.9, 532.0-532.9, 533.0-533.9, 534.0-534.9 |
| Liver disease | 571.2, 571.4, 571.5, 571.6, 572.2-572.8, 456.0-456.21 |
| Diabetes | 250.0-250.3, 250.4, 250.5, 250.6, 250.7, 250.8, 250.9 |
| Hemiplegia or paraplegia | 342, 344.1 |
| Renal disease | 582, 583, 585, 586, 588 |
| Neoplastic disease | 140-172, 174-208 |
| HIV/AIDS | 42-44, V08 |
| Bacteremia | 790.7 |
| Pneumonia | 480.0-483.99, 485–487 |
| Skin infection | 680-686 |
| Endocarditis | 421.0, 421.1, 421.9, 424.9 |
| Urinary tract infection | 590-599 |
| Device-related infection | 996.31, 996.62, 996.64, 999.31 |
| Acute respiratory infection | 460-466 |
| GERD | 530.11, 530.81 |
| Transplant | V42, E878.0 |
| Inflammatory bowel disease | 555, 556 |
| Irritable bowel syndrome | 564.1 |
| **Concomitant infections** | |
| Bacteremia | 790.7 |
| Pneumonia | 480.0-483.99, 485–487 |
| Skin infection | 680-686 |
| Intra-abdominal infection | 540–543, 562, 567, 569, 574–577 |
| Urinary tract infection | 590-599 |
| Device-related infection | 996.31, 996.62, 996.64, 999.31 |
| Acute respiratory infection | 460-466 |
| Endocarditis | 421.0, 421.1, 421.9, 424.9 |
| **CDI severity indicators** | |
| Shock | 639.5, 785.52, 785.59 |
| Sepsis/septicemia | 020.2, 038.0-038.9, 995.91, 995.92 |
| Perforation of intestine | 569.83 |
| Prolonged ileus | 560.1 |
| Megacolon | 558.2, 564.7 |
| Acute renal failure | 584, 586 |
| Colectomy | 45.73, 45.81-83 (procedure codes) |
